# Common pitfalls in critical care research

**DOI:** 10.62675/2965-2774.20250339

**Published:** 2025-04-16

**Authors:** Bruno Adler Maccagnan Pinheiro Besen, Antônio Paulo Nassar, Juliana Carvalho Ferreira, Otavio Ranzani

**Affiliations:** 1 Instituto D’Or de Pesquisa e Ensino São Paulo SP Brazil Instituto D’Or de Pesquisa e Ensino - São Paulo (SP), Brazil.; 2 Universidade de São Paulo Faculdade de Medicina Department of Internal Medicine São Paulo SP Brazil Postgraduate Program in Medical Sciences, Department of Internal Medicine, Faculdade de Medicina, Universidade de São Paulo - São Paulo (SP), Brazil.; 3 Intensive Care Unit A.C. Camargo Cancer Center São Paulo SP Brazil Intensive Care Unit, A.C. Camargo Cancer Center - São Paulo (SP), Brazil.; 4 Universidade de São Paulo Instituto do Coração Faculdade de Medicina São Paulo SP Brazil Division of Pulmonology, Instituto do Coração, Hospital das Clínicas, Faculdade de Medicina, Universidade de São Paulo - São Paulo (SP), Brazil.; 5 Barcelona Institute for Global Health Barcelona Spain Barcelona Institute for Global Health - Barcelona, Spain.

## INTRODUCTION

Critical care research is characterized by many opportunities due to the data-rich environment of the intensive care unit (ICU): physiological monitoring derangements, patients’ prior characteristics, acute organ dysfunction, metabolic derangements, and many laboratory and organ support measurements. Many ICUs integrate, in real-time, monitors, laboratory results and charts, allowing high time-resolution and completeness, providing large, open-sourced, databases.^(1,2)^ While this leads to a scientifically rich environment, it may cloud scientific thinking and lead to data-driven studies without clear hypotheses. Here, we will discuss common pitfalls researchers should be aware of when conducting critical care research.

### Lack of a clear, relevant research question

A clear research question is a tenet for the planning, execution, and interpretation of the results, and it will pave the way to avoid many pitfalls. The underlying theory must guide the research, not the statistical results. Although it seems trivial, even experienced researchers may dive into a loophole due to an unclear research question. Research question clarity involves two main issues. First, the researcher must be sure of their primary aim: is it mainly descriptive, causal, or predictive? Second, the researcher should have a clear theoretical model of how the intervention works, in case of causal questions, or how a new diagnostic or predictive marker will fit in clinical practice, in case of diagnostic and prognostic research. Mixing concepts is very common and clouds scientific thinking.

### Definition of inclusion and exclusion criteria

In clinical trials, much thought is put into inclusion and exclusion criteria to adequately sample the target population. This rationale must be followed for other study designs too and it is dependent on the study aim: for causal questions, exclusion criteria should mirror those of a pragmatic clinical trial. For prognosis and diagnosis questions, the study population should mirror the one that would have the test applied in practice. Authors should avoid excluding patients due to missing data, which should be appropriately dealt with.^(3)^

### Over reliance on statistical significance instead of estimation

Traditional teaching in healthcare research emphasizes null hypothesis significance testing and p value interpretation. Researchers must focus, however, on estimation.^(4)^ Quantitative research is about estimating a given quantity of interest. It could be the prevalence of a condition for descriptive research, an estimate of risk ratios for a causal question, or the risk of an outcome for prognosis studies. This does not mean statistical significance is doomed, but that it must be presented with point estimates and confidence intervals and, sometimes, not even conducted depending on the scenario.^(5)^

### Association of physiological derangements with death

Although critical care practice is intimately related to avoiding death, authors should avoid the common shortcut of finding an association between physiological derangements and mortality. Essentially, all physiological derangements will be to some extent associated with mortality (i.e., those who are most seriously ill are more likely to die), but these are commonly only prognostic associations. Well-thought research questions may need a demonstration of this association at the outstart of a line of research, but authors should consider potential causal paths amenable to treatment when moving forward.

### Variable selection for multivariable models

Variable selection must be aligned with the research question. For causal questions, variable selection should be based on subject-matter knowledge by drawing conceptual models, such as causal directed acyclic graphs (DAGs).^(6,7)^ For predictive studies, as the aim is to estimate a probability, the causal relationship is not essential, but the outcome explainability.^(8)^ Nevertheless, for predictive questions, the authors must consider what variables are available at the point of care. These variables should not be a surrogate of what is meant to be predicted, which would lead to overly optimistic estimates of the model and poor applicability. Mixing concepts of prediction variable selection with DAG-based variable selection for causal questions may lead to low-value data presentation.

### Table 2 Fallacy

The Table 2 Fallacy is an underrecognized issue in observational studies with multivariable models.^(9)^ Except in particular circumstances of prognostic research in which researchers aim to find an association of a combination of variables with clinical outcomes, not all coefficients of a multivariable model should be presented. In causal research, variables included in a multivariable model for adjustment should not have their results presented since *their* "effect estimate" is not estimated by *that* model. Each set of variables included in a model was so because they were necessary to address that research question. Furthermore, even in prognostic research, the association of a new prognostic variable with outcome should be evaluated by accounting for known, observed prognostic variables in the model, regardless of hypothesis testing in that given dataset.

### No pre-registration

Registration of clinical trials is a standard practice to avoid publication bias and undescribed modifications to study procedures during study roll-out. Systematic reviews must also have a registered protocol to allow *a-priori* hypothesis specification.^(10)^ Protocol deviations must be justified when needed. Absent pre-registration might preclude their publication. While observational studies still do not need pre-registration, authors should consider doing so or making the analysis protocol publicly available.

### Abstracts without estimates

Abstracts are the first impression of a manuscript. In an abstract, a single main clear objective should be answered with the conclusion of the manuscript, but the focus should be on a brief description of methods and the main results’ presentation. The results need to be presented with estimates and their confidence intervals. Authors should avoid presenting only p values.

### New methods are not always better methods

While as scientists we desire better methods to answer our research questions, new methods are not necessarily better methods. For example, the net reclassification improvement arose a decade ago as a new method for biomarker research, but it did not pass the test of time.^(11)^ While Bayesian analysis,^(12)^ machine learning, and artificial intelligence^(13,14)^ are in the spotlight, they should not drive the research question.

## CONCLUSION

**Figure 1:**
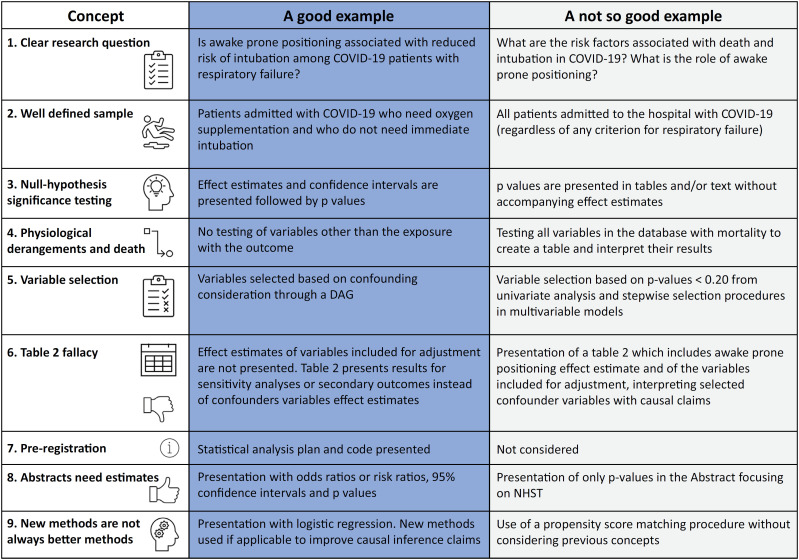
Examples of a contrast of a good (and not so good) hypothetical observational study about the effects of awake prone positioning in hospitalized COVID-19 patients with respiratory failure. DAG - directed acyclic graph; NHST - null hypothesis significance testing.

From this viewpoint, we presented a few issues that we believe are important for critical care researchers. Figure 1 provides a contrast of good *versus* not-so-good practices in an observational study for further reference, which can be applied to a recently published systematic review.^(15)^
